# Incidental findings in the oncology patient: liver

**DOI:** 10.1186/1470-7330-14-S1-O42

**Published:** 2014-10-09

**Authors:** Wolfgang Schima

**Affiliations:** 1 Department of Diagnostic and Interventional Radiology, Krankenhaus Goettlicher Heiland, KH der Barmherzigen Schwestern, and Sankt Josef-Krankenhaus, Vinzenzgruppe, Dornbacher Strasse 20-28, 1170 Vienna, Austria

## 

Technical advances in ultrasound, multi-detector computed tomography (MDCT) and magnetic resonance imaging (MRI) have increased our ability to detect small-sized hepatic lesions and low-contrast lesions, which would have escaped detection some years ago [1,2]. Prevalence of small lesions found at CT ranges from 12.7% to 29.4% in cancer patients [1-3]. Only a minority of these lesions will eventually turn out to be malignant. However, these incidental findings (or “incidentalomas”, as they are called) detected in an oncologic patient pose a particular challenge for both the reporting radiologist and the referring clinician. Contrast-enhanced MRI has additional value in characterization of small lesions indeterminate at CT [4,5], but it has to be justified in terms of cost and resources in an individual patient. Management strategies are being developed to address these lesions, whether aggressive further evaluation (including contrast-enhanced MRI and/or biopsy) or imaging follow-up is sought [6]. Seeking a “100%-certainty strategy” may result in unnecessarily costly and invasive work-up of many patients. Decisions on further management should depend on the imaging appearance of incidentalomas, the history of the patients, risk assessment, taking into account further treatment options.

Imaging appearance of focal fatty infiltration and focal sparing of fat, which may mimic malignant disease, is presented. Small benign lesions, such as flash-filling hemangiomas, FNH, biliary hamartomas, or solitary necrotizing nodules can be difficult to diagnose in the setting of primary tumors with either hypervascular or hypovascular metastases, respectively. Strategies to approach these lesions and guide further management are presented.
